# Lactic Acid Bacteria Isolated from the Microflora and Silage of *Agropyron* spp. as Bio-Inoculants for Difficult-to-Ensile Forage Crops

**DOI:** 10.3390/microorganisms14071460

**Published:** 2026-07-02

**Authors:** Raushan Zh. Kaptagai, Gani K. Taubekova, Zhanar Sh. Zhumadilova, Akbota T. Tassyrbayeva, Amankeldi K. Sadanov, Yerik Zh. Shorabaev, Karlygash M. Abdiyeva

**Affiliations:** 1 Industrial Microbiology LLP., Almaty 050060, Kazakhstan; kaptagaeva_raushan@mail.ru (R.Z.K.); tgaini.61@gmail.com (G.K.T.); eshorabaev@mail.ru (Y.Z.S.); 2 Department of Biochemical Engineering, International Engineering and Technological University, Almaty 050060, Kazakhstan; a.tasyrbaeva@mail.ru; 3 Research and Production Center of Microbiology and Virology, Almaty 050010, Kazakhstan; a.sadanov1951@gmail.com; 4 Department of Food Technology, Almaty Technological University, Almaty 050012, Kazakhstan; k.abdiyeva08@gmail.com

**Keywords:** wheatgrass (*Agropyron* spp.), lactic acid bacteria, screening, identification, silage

## Abstract

The aim of this study was to isolate and molecularly identify lactic acid bacteria (LAB) associated with the epiphytic microflora and silage of wheatgrass (*Agropyron* spp.), as well as to evaluate their biotechnological potential as starter cultures for the ensiling of difficult-to-ensile forage crops under the climatic conditions of northern Kazakhstan. A total of 63 bacterial isolates were obtained and grown on MRS medium under different temperature conditions. Based on growth characteristics, pH values, and titratable acidity, 15 highly active strains were selected, demonstrating stable acidification (pH 3.99–4.75) and high metabolic activity. All isolates were catalase negative and capable of fermenting a wide range of carbohydrates and polyols, although pronounced strain-specific differences were observed. The selected strains exhibited proteolytic and antagonistic activity against test microorganisms and showed high tolerance to osmotic stress, maintaining growth at NaCl concentrations of up to 8–10%. Molecular identification based on 16S rRNA gene sequencing revealed that nine technologically significant strains belonged to the species *Lactococcus garvieae*, *Pediococcus acidilactici*, *Lactiplantibacillus plantarum*, *Enterococcus faecalis* and *Enterococcus faecium*. The results obtained in this study demonstrate the high environmental adaptability of the isolated strains and confirm their potential for the development of effective microbial inoculants aimed at improving fermentation processes and enhancing the preservation of difficult-to-ensile forage crops under cold-climate conditions.

## 1. Introduction

Livestock production is a strategically important sector of agriculture in Kazakhstan, and the efficiency of animal production largely depends on the quality of the feed base and the forage preservation technologies applied. In the northern regions of Kazakhstan, characterized by a harsh continental climate, a short growing season, limited moisture availability, and frequent temperature fluctuations, the cultivation of resilient forage crops and the development of effective preservation technologies are of particular importance [1].

Perennial grasses constitute a significant proportion of forage lands in northern Kazakhstan, among which wheatgrass (*Agropyron* spp.) occupies an important position. This crop is characterized by high drought and frost tolerance, environmental adaptability, and the ability to produce stable biomass under conditions of limited heat and moisture availability. Currently, wheatgrass is mainly used for hay production; however, the drying process is associated with considerable losses of nutrients, including proteins, water-soluble carbohydrates, and vitamins. Therefore, ensiling is considered a more efficient forage preservation method that allows better retention of the nutritional value of plant biomass.

In recent years, biological preservatives based on LAB have been widely applied in forage crop ensiling. These microorganisms promote rapid acidification of the ensiled biomass through lactic acid production, inhibit the growth of undesirable microflora, and contribute to the stabilization of the fermentation process [2,3]. The application of LAB improves silage quality, reduces dry matter losses, and enhances the aerobic stability of the final feed [4,5,6].

Silage prepared without bacterial inoculants is often characterized by elevated ammonia nitrogen levels, low lactic acid concentration, and an unstable microbial community structure, ultimately leading to reduced nutritional value [4,7].

However, many perennial grasses are classified as difficult-to-ensile crops due to their low content of water-soluble carbohydrates, high buffering capacity, and insufficient populations of naturally occurring epiphytic LAB. This issue is particularly relevant in northern regions with reduced temperatures, where natural fermentation processes occur less intensively. Under such conditions, the use of indigenous LAB strains adapted to specific environmental conditions and possessing greater competitive ability than universal commercial inoculants is considered a promising approach.

Recent studies have shown that representatives of the genera *Lactiplantibacillus*, *Pediococcus*, *Enterococcus*, and *Weissella* are among the dominant members of the epiphytic microflora of silage [8,9,10]. Particular attention has been paid to *Lactiplantibacillus plantarum* and *Pediococcus acidilactici* [11,12,13], which are characterized by high acidification activity, tolerance to environmental stress factors, and the ability to maintain fermentation activity under low-temperature conditions [14,15,16,17].

Recent review studies emphasize that the current trend in silage biotechnology is shifting from the use of universal commercial inoculants toward regionally adapted bacterial consortia based on indigenous strains capable of competing effectively under specific environmental conditions [18].

Despite the considerable number of studies focused on the isolation and application of LAB for the ensiling of natural forages in different regions of the world, information regarding the composition of the epiphytic microflora and the biotechnological potential of indigenous LAB associated with wheatgrass (*Agropyron* spp.) in the northern regions of Kazakhstan remains extremely limited. In particular, systematic studies aimed at the isolation, identification, and selection of indigenous LAB strains with potential application in the ensiling of this crop are lacking in the available literature [19].

Therefore, the aim of the present study was to isolate, screen, and comprehensively characterize LAB associated with the epiphytic microflora of wheatgrass and wheatgrass silage in order to identify promising strains with biotechnological potential for the ensiling of difficult-to-ensile forage crops under the climatic conditions of northern Kazakhstan.

## 2. Materials and Methods

### 2.1. Sampling and Isolation of Lactic Acid Bacteria

Wheatgrass (*Agropyron* spp.) plant material was collected in July 2025 in the Kamysty District of the Kostanay Region (northern Kazakhstan) at the late vegetation stage. Samples were transported in sterile vacuum-sealed bags at 4 °C and delivered to the laboratory within 48 h.

LAB were isolated from the epiphytic microflora of wheatgrass (*Agropyron* spp.), including leaves, stems, spikes, seeds, roots, and whole-plant samples, as well as from silage prepared from forage crops. In total, 63 LAB isolates were obtained, including 33 strains isolated from the epiphytic plant microflora: 5 from stems, 5 from seeds, 5 from spikes, 5 from leaves, 4 from roots, and 9 from whole plants. In addition, 30 strains were isolated from silage samples.

For the isolation of epiphytic LAB, 10 g portions of plant material and silage samples were aseptically transferred into flasks containing 90 mL of sterile distilled water. The samples were homogenized for 15–20 min on an orbital shaker at room temperature, followed by serial tenfold dilutions.

Isolation and cultivation of LAB were performed using modified de Man–Rogosa–Sharpe (MRS) medium in liquid, semi-solid (0.07% agar), and solid (2% agar) forms. The composition of the MRS medium (g/L) was as follows: meat peptone broth, 5.0; peptone, 20.0; yeast extract, 5.0; glucose, 20.0; KH_2_PO_4_, 2.0; sodium acetate, 5.0; ammonium citrate, 2.0; MgSO_4_, 0.1; MnCl_2_, 0.05; final pH 7.0 [20].

Diluted suspensions were spread onto Petri dishes containing solid MRS medium and incubated under anaerobic conditions at 35–38 °C for 48 h. Anaerobic conditions were established using anaerobic jars and gas-generating sachets.

After incubation, colonies differing in morphological characteristics (shape, size, color, surface morphology, and colony margins) were selected. Pure cultures were obtained by repeated streaking on MRS agar plates.

For long-term preservation, isolates were cultivated in liquid MRS medium supplemented with 25% sterile glycerol and stored at −80 °C.

Preliminary screening of active strains was performed based on their acidification capacity during cultivation in liquid MRS medium. Acidification activity was evaluated by measuring changes in pH and titratable acidity (°T).

Microscopic examination of bacterial cells was carried out using a laboratory microscope equipped with a 4-megapixel EX30 camera (Ningbo Sunny Instruments Co., Ltd., Ningbo, China).

### 2.2. Physiological and Biochemical Characterization

Carbohydrate fermentation was determined using Giss medium supplemented with Andrade indicator according to standard protocols [21]. A total of 16 substrates were evaluated, including glucose, xylose, fructose, maltose, arabinose, lactose, raffinose, ribose, rhamnose, mannose, cellulose, hemicellulose, starch and mannitol. The medium was dispensed into 10 mL tubes containing inverted Durham tubes for gas detection. Tubes were incubated at 35–37 °C for 18–48 h. Acid production was determined by a color change of the medium to red, whereas gas formation was assessed by gas accumulation in Durham tubes.

Gram staining was performed according to standard microbiological procedures. Catalase activity was evaluated by adding 0.1–0.2 mL of 3% hydrogen peroxide to 2–3 mL of bacterial suspension grown in MRS broth and observing bubble formation.

Acidification activity was determined based on titratable acidity expressed in Turner degrees (°T). The pH values were measured using an electrochemical analyzer pH meter (Consort C931, B-2300 Turnhout, Belgium). All experiments were performed in three independent replicates. The results are presented as mean values ± standard deviation (Mean ± SD).

Nitrogen source utilization was evaluated using a synthetic medium containing the following components (g/L): NaH_2_PO_4_, 1.0; K_2_HPO_4_, 1.0; MgSO_4_, 1.0; sucrose, 1.0; and CaCO_3_, 20.0. Peptone, ammonium sulfate, urea, and yeast autolysate were separately used as nitrogen sources at different concentrations.

Proteolytic activity of LAB was evaluated using skim milk as a substrate. The milk was boiled twice, cooled, and the surface film formed during cooling was removed. Subsequently, the milk was diluted with tap water and dispensed into sterile tubes (10 mL per tube). Sterilization was performed at 0.5 atm pressure.

Bacterial suspensions of the tested strains were inoculated at a volume of 0.1 mL and incubated at 36 °C for 2–10 days. Proteolytic activity was assessed based on the ability of the strains to induce milk coagulation and peptonization.

The ability of the strains to hydrolyze gelatin was determined using meat-peptone gelatin (MPG) medium. The medium was prepared by adding 10–15 g of gelatin to 100 mL of meat-peptone broth (MPB), followed by swelling for 20–30 min. After swelling, the mixture was heated in a water bath until complete dissolution of gelatin, dispensed into sterile tubes (10 mL per tube), and sterilized at 0.5 atm pressure for 15 min.

The tested strains were inoculated into MPG tubes and incubated at room temperature for 7–10 days. Gelatinase activity was evaluated visually based on the presence or absence of gelatin liquefaction.

### 2.3. Antagonistic Activity

Antagonistic activity was evaluated using the agar well diffusion method. Agar plates inoculated with test microorganisms (*Bacillus subtilis*, *Pasteurella multocida*, *Pseudomonas* sp., *Candida albicans*, *Fusarium solani*, *Aspergillus niger*, and *Botrytis cinerea*) were prepared, and wells with a diameter of 8 mm were formed in the agar [22]. The wells were filled with LAB cultures, and the plates were incubated at 37 °C for 24 h. The cell density of the inoculated cultures was approximately 10^9^ CFU/mL. The inoculum density corresponded to a 0.5 McFarland standard [23].

Antimicrobial activity was assessed by measuring the diameter of the growth inhibition zones surrounding the wells. Measurements were performed in three independent replicates, and the results were expressed in millimeters as mean values ± standard deviation (Mean ± SD).

### 2.4. Temperature and Salt Tolerance

The growth of the isolates was evaluated at 20, 26, 37, and 45 °C, as well as on MRS agar supplemented with NaCl at concentrations of 2%, 4%, 6%, 8%, and 10%. Cultures were incubated at 35 °C for 120 h.

Strain growth was assessed based on acidification capacity, changes in medium pH, and visual growth assessment. All experiments were performed in three biological replicates.

### 2.5. Statistical Analysis

All experiments were performed in three independent replicates. Data are presented as mean ± standard deviation (M ± SD) or as median values, depending on the characteristics of the analyzed parameter.

The effects of cultivation temperature on pH and titratable acidity of each strain, as well as the effects of NaCl concentration on bacterial growth, pH, and titratable acidity, were evaluated using the non-parametric Kruskal–Wallis test. Antagonistic activity was assessed by comparing the diameters of inhibition zones produced by different lactic acid bacterial strains against each test culture separately (*Pseudomonas* sp., *Pasteurella multocida*, *Bacillus subtilis*, *Candida albicans*, and *Aspergillus niger*). When significant differences were detected, pairwise comparisons were performed using Dunn’s post hoc test with Bonferroni correction.

Differences were considered statistically significant at *p* < 0.05. In tables and figures, different letter superscripts (a, b, c) indicate statistically significant differences between the compared groups, whereas identical letters indicate the absence of significant differences. The designation “ab” represents an intermediate group that does not differ significantly from groups labeled “a” or “b”.

### 2.6. DNA Extraction and 16S rRNA Gene Sequencing

Genomic DNA was extracted using the GeneJET Genomic DNA Purification Kit (Thermo Fisher Scientific, Waltham, MA, USA) according to the manufacturer’s instructions. For Gram-positive bacteria, a lysis buffer containing 20 mM Tris–HCl (pH 8.0), 2 mM EDTA, 1.2% Triton X-100, and 20 mg/mL lysozyme was used. Bacterial cell pellets were incubated in the lysis buffer for 30 min at 37 °C, followed by proteinase K treatment and column purification. DNA was eluted in 200 μL of elution buffer and stored at −20 °C.

DNA concentration and purity were determined using a NanoDrop ND-2000 spectrophotometer (Thermo Fisher Scientific, Waltham, MA, USA) at 260 nm, while DNA integrity was verified by electrophoresis in a 1% agarose gel.

The 16S rRNA gene was amplified using the universal primers 27F (5′-AGAGTTTGATCCTGGCTCAG-3′) and 1492R (5′-TACGGTTACCTTGTTACGACTT-3′) [24]. The PCR reaction mixture (20 μL) contained up to 30 ng of DNA, 5 U of Taq polymerase, 0.2 mM dNTPs, 10× Taq buffer (Thermo Fisher Scientific, Waltham, MA, USA), 2.5 mM MgCl_2_, and 10 pmol of each primer. Amplification was performed using a ProFlex thermal cycler (Applied Biosystems, Thermo Fisher Scientific, Waltham, MA, USA) under the following conditions: initial denaturation at 95 °C for 5 min; 30 cycles of 95 °C for 30 s, 55 °C for 40 s, and 72 °C for 50 s; followed by a final extension at 72 °C for 10 min.

PCR products were visualized on a 1% agarose gel stained with ethidium bromide. Amplicons were purified enzymatically using exonuclease I and shrimp alkaline phosphatase and subsequently sequenced using BigDye^®^ Terminator v3.1 chemistry [25].

The obtained sequences were compared with reference sequences available in the GenBank database (http://www.ncbi.nlm.nih.gov/, accessed on 24 June 2026) [26]. Phylogenetic analysis was performed using MEGA 11 software [27]. The analysis included 16S rRNA gene nucleotide sequences of the most closely related phylogenetic taxa. Multiple sequence alignment was carried out using the ClustalW algorithm, and phylogenetic trees were constructed using the Neighbor-Joining (NJ) method.

## 3. Results

The screening results demonstrated that the isolated strains differed in their morphological, physiological, and biochemical characteristics. Among the isolates obtained from different parts of wheatgrass, Gram-positive rod-shaped and coccoid bacteria predominated, occurring singly, in pairs, or in short chains.

The colonies were predominantly circular with smooth margins, cream to milky-white coloration, and diameters ranging from 1 to 6 mm. Some strains formed convex translucent colonies.

Isolates obtained from silage samples were mainly represented by small Gram-positive rod-shaped bacteria forming cream-white translucent colonies. All tested cultures were non-motile and catalase negative.

Further screening of the 63 isolates resulted in the selection of 15 strains (1/1, 4/2, 5/3, 8/4, 12/5, 25/11, 26/6, 27/7, 28/8, 29/9, 30/10, 39/12, 41/13, 46/14, and 48/15) characterized by stable pH values (3.99–4.75) and high acidification activity under different temperature conditions (Table 1).

All selected strains were identified as Gram-positive and catalase-negative bacteria.

The pH values and titratable acidity of the investigated lactic acid bacterial strains cultured at different temperatures are presented in Table 1.

Cultivation temperature significantly affected the acid-producing activity of most of the strains studied. Overall, the greatest acidification of the medium and the highest titratable acidity values were observed at 37 °C. For most strains, statistically significant differences were primarily associated with this temperature regime, whereas the parameters measured at 20 and 26 °C generally did not differ significantly.

The lowest pH value was recorded for strain 5/3 at 37 °C (3.99 ± 0.02), whereas the highest titratable acidity was observed for strain 29/9 at the same temperature (150.0 ± 2.0 °T). In general, several strains exhibited the most intensive acid accumulation at 37 °C, which was accompanied by a decrease in pH and an increase in titratable acidity.

A statistically significant effect of cultivation temperature on both pH and titratable acidity was detected for strains 4/2, 5/3, 25/11, 26/6, 27/7, 28/8, 29/9, 30/10, 39/12, and 48/15. For strains 8/4 and 41/13, significant differences were observed only for pH, whereas changes in titratable acidity did not reach statistical significance (*p* > 0.05). In contrast, strains 1/1, 12/5, and 46/14 exhibited stable pH and titratable acidity values across the entire temperature range studied (*p* > 0.05).

These findings indicate that 37 °C was the most favorable temperature for the expression of the acidification potential of the majority of the investigated lactic acid bacterial strains, whereas this effect was less pronounced at 20, 26 and 45 °C.

To evaluate the ability of the strains to utilize carbohydrates and polyols, the fermentation of 14 substrates was assessed, including glucose, xylose, fructose, maltose, lactose, raffinose, ribose, arabinose, rhamnose, mannose, cellulose, hemicellulose, starch, and mannitol.

All 15 selected strains were capable of fermenting the majority of the tested substrates (Table 2). The most intensive fermentation activity was observed for glucose, xylose, fructose, ribose, and mannose, accompanied by a decrease in medium pH.

Strain 39/12 was unable to ferment cellulose, hemicellulose, starch, or mannitol. Several strains exhibited weak fermentation activity toward raffinose, rhamnose, cellulose, hemicellulose, starch, and mannitol.

All selected strains fermented glucose without gas production, which is characteristic of LAB.

**Figure 1:**
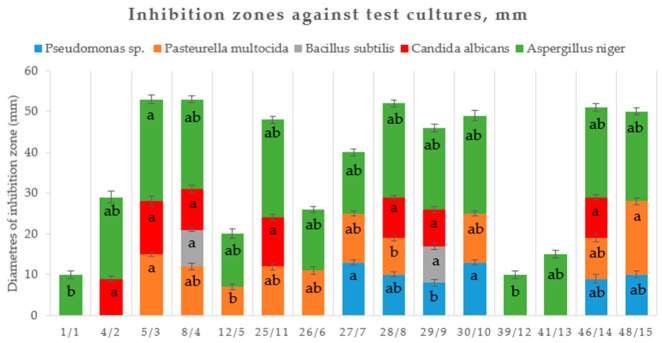
Antagonistic activity of the selected strains. Note. Different lowercase letters indicate statistically significant differences among strains within the same test culture according to the Kruskal–Wallis test followed by Dunn’s post hoc test (*p* < 0.05).

In addition, the antagonistic properties of the selected LAB strains against opportunistic microorganisms and microscopic fungi were evaluated (Figure 1).

The antagonistic activity of the investigated *Bacillus* spp. strains varied considerably depending on the test culture (Figure 1).

The strongest inhibitory activity was observed against *Aspergillus niger*. The inhibition zone diameters ranged from 10.0 ± 1.0 mm (strains 1/1 and 39/12) to 25.0 ± 1.0 mm (strain 5/3). According to the Kruskal–Wallis test, the differences among strains were statistically significant (χ^2^ = 41.53, df = 14, *p* < 0.001). Strain 5/3 exhibited significantly stronger inhibition of *A. niger* than strains 1/1 and 39/12 (*p* < 0.001 for both comparisons).

Only 7 of the 15 strains inhibited the growth of *Candida albicans*. The inhibition zones ranged from 9.0 ± 0.6 mm (strain 4/2) and 9.0 ± 0.8 mm (strain 29/9) to 13.0 ± 1.3 mm (strain 5/3). No significant differences in inhibition zone diameters were detected among the antagonistically active strains.

For *Pasteurella multocida*, inhibition zone diameters ranged from 7.0 ± 0.7 mm (strain 12/5) to 18.0 ± 0.9 mm (strain 48/15). Strains 5/3 and 48/15 exhibited significantly greater antagonistic activity than strains 12/5 and 28/8.

Against *Pseudomonas* sp., inhibitory activity was observed only in six strains and ranged from 8.0 ± 0.9 mm (strain 29/9) to 13.0 ± 0.7 mm (strain 27/7) and 13.0 ± 0.6 mm (strain 30/10). Strains 27/7 and 30/10 inhibited the growth of the test culture significantly more strongly than strain 29/9 (*p* < 0.0033 for both comparisons).

Antagonistic activity against *Bacillus subtilis* was detected only in strains 8/4 (9.0 ± 0.5 mm) and 29/9 (9.0 ± 0.8 mm).

No antagonistic activity was detected against *Fusarium solani* or *Botrytis cinerea*.

**Figure 2:**
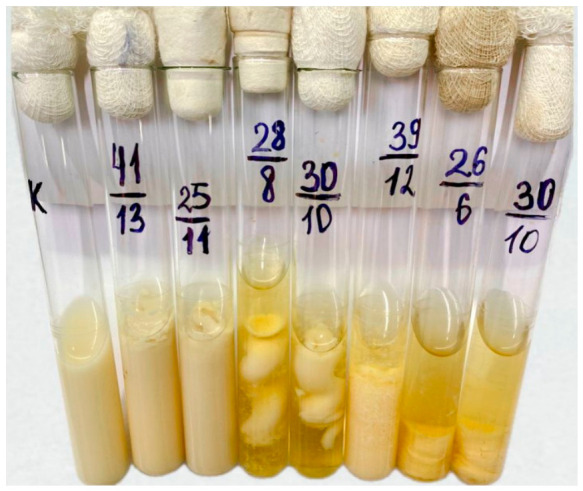
Peptonization of LAB strains.

Evaluation of proteolytic activity demonstrated that the isolated strains differed in their ability to induce milk coagulation and peptonization (Figure 2). The highest proteolytic activity was observed for strains 26/6, 27/7, 28/8, and 30/10, which showed intensive milk peptonization. Strains 3/5, 8/4, 12/5, 25/11, 39/12, and 48/15 exhibited moderate proteolytic activity.

All investigated strains showed no gelatinase or amylolytic activity and did not produce ammonia (Table 3).

Increasing NaCl concentration had a statistically significant effect on acidity, titratable acidity, and viable cell counts in most of the investigated LAB strains (Table 4, Table 5 and Table 6). However, the magnitude of the response varied considerably among strains.

Significant differences in acidity among NaCl concentrations were detected in 13 of the 14 strains studied (*p* = 0.011–0.028), whereas no significant effect of NaCl was observed for strain 12/5 (*p* = 0.076). In general, the highest acidity values were recorded at NaCl concentrations ranging from 2 to 6%, while a significant decrease was observed at 10% NaCl. Multiple comparison analysis revealed that the main source of variation was the contrast between low (2–4%) and high (10%) salt concentrations. However, intermediate NaCl concentrations formed overlapping statistical groups in strains 8/4 and 27/7, indicating greater resistance of their metabolic activity to osmotic stress.

A similar pattern was observed for titratable acidity. A statistically significant effect of NaCl concentration was detected in all strains except 12/5 (*p* = 0.090). For most strains, titratable acidity remained relatively stable at salt concentrations up to 6%, whereas a substantial decline was observed at 8–10% NaCl. The most pronounced response to salt stress was observed in strains 4/2, 25/11, 39/12, 41/13, 46/14, and 48/15, in which the 10% NaCl treatment formed a distinct statistical group compared with lower salt concentrations. In contrast, strain 27/7 maintained relatively high titratable acidity throughout the entire range of NaCl concentrations tested, indicating enhanced tolerance to elevated osmotic pressure.

The viable cell count (log CFU/mL) showed a similar response pattern. In all investigated strains, NaCl concentration significantly affected cell survival (*p* = 0.009–0.024). For most strains, the highest viable counts were observed at 2–4% NaCl, whereas a marked reduction in cell numbers was recorded at 10% NaCl. The strongest inhibitory effect of high salt concentration was observed in strains 4/2, 26/6, 27/7, 28/8, 29/9, 48/15, 25/11, 39/12, 41/13, and 46/14, where the 10% NaCl treatment differed significantly from the low-salt conditions. For strains 5/3, 12/5, and 30/10, significant differences were detected only between the extreme salinity levels, while intermediate concentrations formed overlapping statistical groups.

Overall, the results indicate that NaCl concentrations up to 6% exerted only a moderate effect on the growth and acid-producing activity of most of the investigated lactic acid bacterial strains. Increasing the salt concentration to 10% consistently reduced both metabolic activity and viable cell numbers, although the degree of inhibition varied substantially among strains. Strains 12/5 and 27/7 exhibited the greatest stability in acidification characteristics, whereas strains 4/2, 39/12, 41/13, 46/14, and 48/15 were the most sensitive to high NaCl concentrations.

The concentration of the extracted DNA ranged from 141 to 328 ng/μL, indicating a sufficient yield for molecular analysis. The A260/280 ratios varied between 1.8 and 2.0, confirming the high purity of the DNA samples (Table 5).

### 3.1. Amplification of the 16S rRNA Gene Fragment

**Figure 3:**
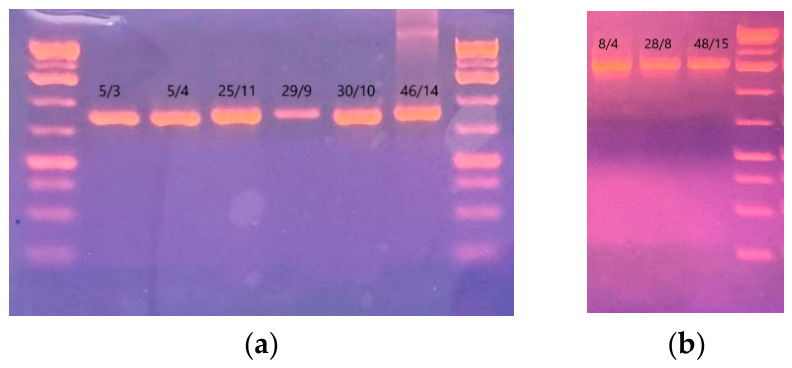
Electropherogram of PCR amplification products of the 16S rRNA gene fragments obtained from nine DNA samples (**a**,**b**). Lane designations (**a**): Lane 1, (M) DNA molecular weight marker GeneRuler 100 bp Plus (100–3000 bp); lanes 2–7, samples 5/3, 5/4, 25/11, 29/9, 30/10, and 46/14. Lane designations (**b**): Lanes 1–3, samples 8/4, 28/8, and 48/15; lane 4, DNA molecular weight marker GeneRuler 100 bp Plus (100–3000 bp).

Successful amplification of the target 16S rRNA gene fragment was observed for all nine isolates, as confirmed by agarose gel electrophoresis (Figure 3). The obtained amplicons produced clear and specific bands.

### 3.2. Taxonomic Identification

BLAST analysis revealed that the isolates belonged to different genera of LAB and associated microorganisms:

- *Lactococcus garvieae* (strain 5/3)
- *Staphylococcus epidermidis*/*Staphylococcus* sp. (strain 5/4)
- *Pediococcus acidilactici* (strains 25/11, 46/14, and 48/15)
- *Lactiplantibacillus plantarum* (strain 29/9)
- *Enterococcus faecalis* (strains 30/10 and 28/8)
- *Enterococcus faecium* (strain 8/4)

Sequence identity values ranged from 98.71% to 100% (Table 6), confirming the reliability of the taxonomic identification.

### 3.3. Phylogenetic Analysis

**Figure 4:**
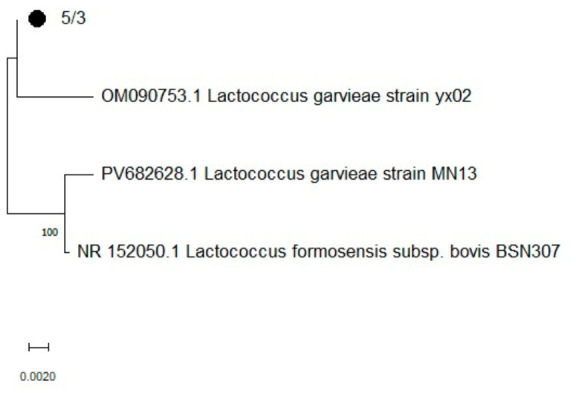
Phylogenetic tree constructed based on the analysis of 16S rRNA gene nucleotide sequences of the genus *Lactococcus*.

**Figure 5:**
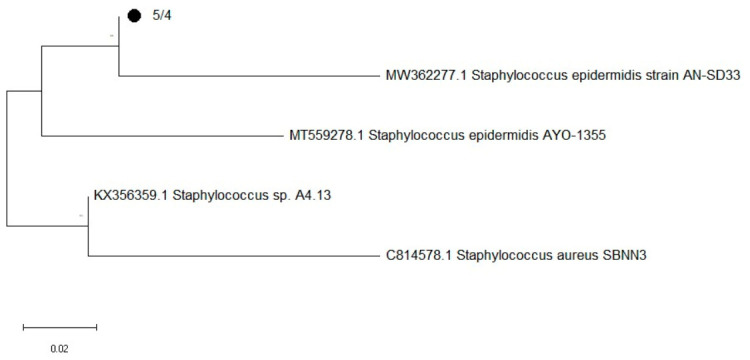
Phylogenetic tree constructed based on the analysis of 16S rRNA gene nucleotide sequences of the genus *Staphylococcus*.

**Figure 6:**
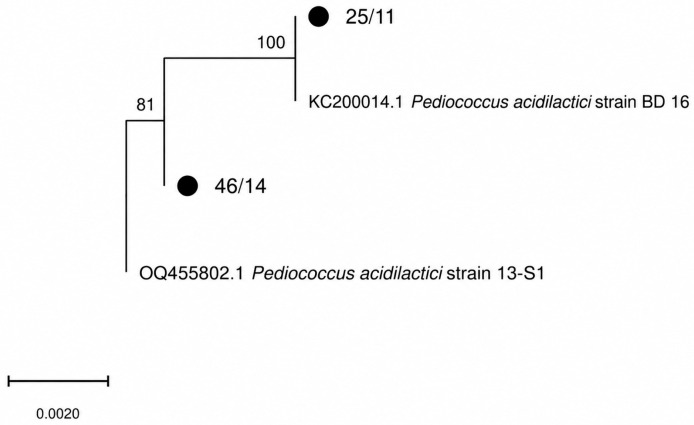
Phylogenetic tree constructed based on the analysis of 16S rRNA gene nucleotide sequences of the genus *Pediococcus*.

**Figure 7:**
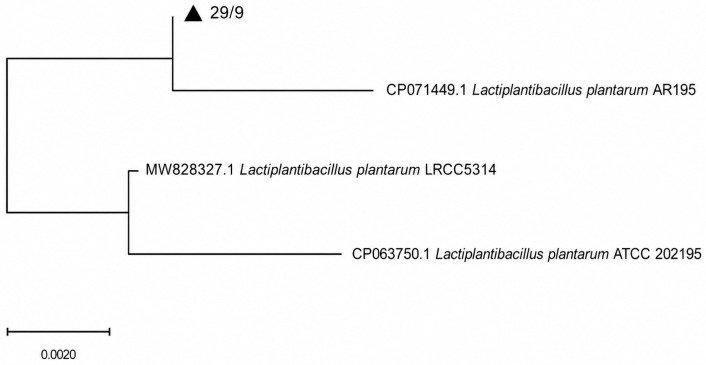
Phylogenetic tree constructed based on the analysis of 16S rRNA gene nucleotide sequences of the genus *Lactiplantibacillus*.

**Figure 8:**
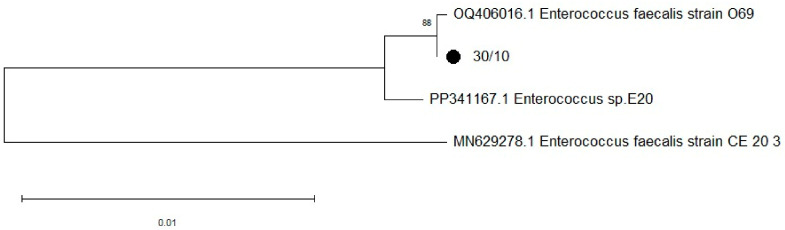
Phylogenetic tree constructed based on the analysis of 16S rRNA gene nucleotide sequences of the genus *Enterococcus*.

**Figure 9:**
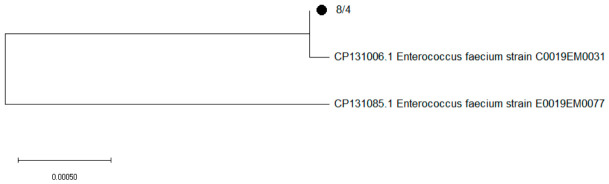
Phylogenetic tree constructed based on the analysis of 16S rRNA gene nucleotide sequences of the genus *Enterococcus*.

**Figure 10:**
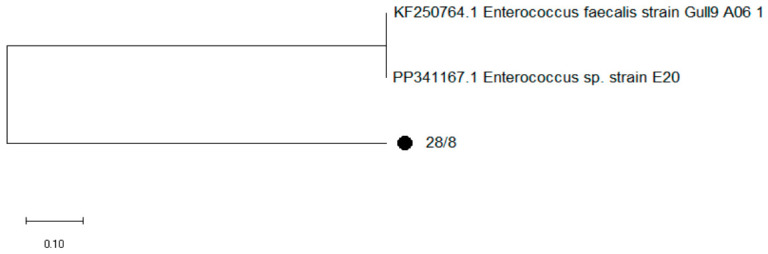
Phylogenetic tree constructed based on the analysis of 16S rRNA gene nucleotide sequences of the genus *Enterococcus*.

**Figure 11:**
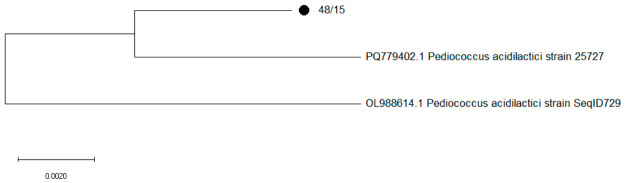
Phylogenetic tree constructed based on the analysis of 16S rRNA gene nucleotide sequences of the genus *Pediococcus*.

The phylogenetic tree constructed using the Neighbor-Joining method confirmed the results of the BLAST analysis. All strains clustered with their corresponding reference species from the GenBank database, demonstrating clear phylogenetic grouping within the genera *Lactococcus*, *Pediococcus*, *Enterococcus*, *Lactiplantibacillus*, and *Staphylococcus* (Figure 4, Figure 5, Figure 6, Figure 7, Figure 8, Figure 9, Figure 10 and Figure 11).

As shown in Figure 4, strain 5/3, identified as *Lactococcus garvieae*, clustered within the same phylogenetic branch as reference strains of *Lactococcus garvieae* retrieved from the GenBank database. Thus, both phylogenetic analysis and BLAST sequence analysis confirmed the identification of strain 5/3 as *Lactococcus garvieae*.

As shown in Figure 5, strain 5/4 clustered within the same phylogenetic branch as *Staphylococcus epidermidis*, *Staphylococcus* sp., and *Staphylococcus aureus*, although it showed the closest genetic relationship to *Staphylococcus epidermidis*.

As shown in Figure 6, strains 25/11 and 46/14, which exhibited the highest sequence identity with *Pediococcus acidilactici* in the GenBank database, clustered within the same phylogenetic branch as reference strains of *Pediococcus acidilactici*.

As shown in Figure 7, strain 29/9, which exhibited the highest sequence identity with *Lactiplantibacillus plantarum* in the GenBank database, clustered within the same phylogenetic branch as reference strains of *Lactiplantibacillus plantarum*.

As shown in Figure 8, strain 30/10, identified as *Enterococcus faecalis*, clustered within the same phylogenetic branch as reference strains of *Enterococcus faecalis* from the GenBank database.

As shown in Figure 9, strain 8/4, identified as *Enterococcus faecium*, clustered within the same phylogenetic branch as reference strains of *Enterococcus faecium* from the GenBank database.

As shown in Figure 10, strain 28/8 clustered within the same phylogenetic branch as *Enterococcus faecalis* and *Enterococcus* sp., although it demonstrated the closest genetic relationship to *Enterococcus faecalis*.

As shown in Figure 11, strain 48/15, identified as *Pediococcus acidilactici*, clustered within the same phylogenetic branch as reference strains of *Pediococcus acidilactici* from the GenBank database.

## 4. Discussion

Although, depending on the stage of the fermentation process, in addition to LAB, representatives of the *Enterobacteriaceae* family, aerobic spore-forming bacteria, *Clostridium*, *Listeria*, acetic acid bacteria, and propionibacteria, mold fungi and yeasts [28] can be detected in silage, this study aimed to isolate only LAB from *Agropyron* spp. epiphytic microbiota and silage, reflecting their characteristic presence in mature silage, ecological adaptation to plant substrates, and competitive advantage under conditions of limited nutrient availability.

Wheatgrass is a difficult-to-ensilage cereal plant with a high fiber content and often a deficiency of readily available sugars. For northern Kazakhstan, where harvesting can occur in cool weather, and self-heating occurs within the silage, the starter culture must have a broad temperature optimum. Analysis of the temperature profiles of the studied isolates (Table 1) revealed clear differences in their biotechnological potential by source of isolation. Isolates obtained from finished silage exhibited pronounced thermo- and psi-tolerance. In particular, isolates 27/7 and 28/8 demonstrated the ability to intensively accumulate biomass and significantly reduce pH to 4.49–4.54 at temperatures as low as 20 °C, which is crucial for the successful preservation of difficult-to-ensilage raw materials (wheatgrass) in the low temperatures of northern Kazakhstan. The high metabolic stability of these isolates under heat stress (45 °C) can ensure the suppression of undesirable microflora during the active self-heating stage of the silage mass. Isolates from silage 25/11 and 29/9 also showed similar pH values at the lowest and highest temperatures. In contrast, isolates obtained from the root system (46/14, 48/15) and the whole plant (4/2) were completely inhibited at 45 °C, which excludes their use as independent starter culture agents. Thus, isolates 27/7, 28/8, 29/9, and 25/11 demonstrated the best performance based on a combination of traits (high acidification rate at low temperatures and thermal stability). Other researchers have also demonstrated the ability of LAB intended for silage to grow over a wide temperature range. Thus, You et al. [29] showed that *Lactiplantibacillus plantarum* from native grass silage on the Inner Mongolian Plateau could grow normally at 15–30 °C. In our study, we identified several microorganisms capable of growth and actively acidify the substrate over a wider temperature range—from 20 °C to 45 °C. In turn, in the studies of Bao et al. [30], the temperature range was somewhat wider (15–45 °C) than in our research. However, our selected strains showed higher salt tolerance.

The ability of the investigated strains to maintain growth and acidification activity over a temperature range of 20–45 °C may be associated with the stability of key glycolytic enzymes and the lactate dehydrogenase pathway, which ensure efficient cellular energy metabolism under fluctuating temperatures. In addition, adaptive modifications of membrane lipid composition may contribute to maintaining cellular integrity under stress conditions. Similar adaptive characteristics have previously been reported for indigenous LAB isolated from grassland ecosystems of Inner Mongolia and northern China, where local strains exhibited greater stress tolerance than commercial cultures [4,8].

An important technological characteristic of the selected strains is their ability to rapidly acidify the growth medium while maintaining metabolic activity. This process is associated with the predominance of the homofermentative carbohydrate metabolism pathway, in which lactic acid serves as the major end product. Rapid and sustained pH reduction plays a crucial role in suppressing undesirable microorganisms during the early stages of ensiling [31], primarily through disruption of membrane transport systems and enzymatic activity in acid-sensitive microorganisms. The ability of the investigated strains to remain metabolically active at reduced temperatures further supports their potential application in cold-climate regions, where fermentation efficiency is often limited by reduced metabolic rates. Similar observations have been reported for *Lactiplantibacillus plantarum* during forage ensiling, where this species maintained high fermentation activity under low-temperature conditions [12,17].

Analysis of carbohydrate metabolism revealed that most strains possessed a broad spectrum of fermentative activity toward mono- and disaccharides, including glucose, fructose, lactose, and maltose. This capability may be associated with the presence of active phosphotransferase transport systems (PTS) and inducible hydrolytic enzymes that facilitate adaptation to diverse plant-derived substrates. At the same time, the variable ability of certain isolates to utilize more complex carbohydrates, such as cellulose, hemicellulose, raffinose, and starch, may reflect strain-specific expression of genes encoding the corresponding enzymatic systems and highlights the functional diversity of natural LAB populations. Similar characteristics have previously been reported for representatives of the genera *Pediococcus* and *Enterococcus* isolated from silage and natural plant ecosystems [14,32].

All investigated strains fermented glucose without gas production, indicating a homofermentative type of metabolism. This fermentation pathway is considered technologically advantageous for silage production because it promotes efficient lactic acid accumulation while minimizing dry matter losses [18] due to the absence of by-product formation, such as CO_2_ and other volatile compounds.

For wheatgrass preservation in northern Kazakhstan, suppression of molds (*Aspergillus niger*) and yeasts (*Candida albicans*) is critical, as they cause secondary spoilage and heating of silage when trenches are opened in dry or windy conditions. For its part, the suppression of bacilli (*Bacillus subtilis*) can prevent protein decay. The antagonistic activity observed against *Pseudomonas* sp., *Bacillus subtilis*, *Pasteurella multocida*, and *Candida albicans* may be attributed to the combined effects of organic acid production, pH reduction, competition for available nutrients, and the potential synthesis of bacteriocins and other antimicrobial metabolites. Inhibition of yeast and mold growth by LAB has been previously shown by other researchers [33]. In particular, the LAB species *L. plantarum*, which is characteristic of silage, is known for its suppression of a variety of microorganisms that cause silage spoilage, especially yeasts and mold fungi [34].

Similar mechanisms have been described for *Pediococcus acidilactici* and *Enterococcus faecium*, which are capable of suppressing undesirable microorganisms during the ensiling process [7,16,35]. In contrast, the absence of pronounced activity against *Fusarium solani* and *Botrytis cinerea* suggests a selective pattern of antagonism, potentially associated with differences in fungal tolerance to acidic conditions and antimicrobial compounds. Comparable antimicrobial properties have also been reported for *Lactococcus garvieae*, which contributes to the establishment of a stable microbial ecosystem in silage [13].

Particularly noteworthy was the high salt tolerance exhibited by most strains, which remained viable at NaCl concentrations of 8–10%. This phenomenon may be associated with the activation of osmoregulatory mechanisms, including the accumulation of compatible solutes such as proline and betaine, regulation of ion homeostasis, and adaptive modifications of membrane structure. At the same time, the observed reduction in acidification activity with increasing salt concentration may reflect the redistribution of cellular energy resources toward stress adaptation, resulting in reduced carbohydrate metabolism. Similar responses have previously been reported for LAB exposed to elevated osmotic pressure [14,15]. However, in the studies of You et al. [29], LAB grew only at NaCl concentrations of 3% and 6.5%, while the microorganisms we selected demonstrated much greater resistance. It was found that the strains isolated from the silage mass (28/8, 29/9, 25/11) possess pronounced osmotic tolerance, maintaining a high level of viability (more than 5.0 log CFU/mL) even under conditions of extreme NaCl concentration (10%). Moreover, strain 29/9 demonstrated maximum resistance, maintaining the titer at the level of 6.30 ± 0.18 log CFU/mL. It is important that these strains did not show a critical decrease in titer or blocking of acid-forming capacity with an increase in salt concentration to 6% (pH was maintained within 5.59–5.69), which indicates the stability of their enzymatic systems.

Proteolysis in silage is triggered by both plant enzymes and undesirable bacteria, which break down valuable protein into ammonia and biogenic amines [36]. Assessment of proteolytic activity revealed moderate to high casein hydrolysis by several strains. At the same time, the absence of ammonia production indicates low activity of deaminating enzyme systems, which represents a favorable technological characteristic because it may prevent excessive accumulation of ammonia nitrogen and deterioration of silage quality. Thus, the observed combination of proteolytic activity and the absence of ammonification may be considered advantageous for potential silage applications.

Overall, the study demonstrated the high potential of local strains for developing starter cultures for difficult-to-ensilage crops. The preference for local strains in ensiling has also been emphasized by researchers [37].

Molecular identification based on 16S rRNA gene sequencing revealed the predominance of representatives of the genera *Pediococcus*, *Enterococcus*, *Lactococcus*, and *Lactiplantibacillus*. Among these, *Pediococcus acidilactici*, *Lactiplantibacillus plantarum*, and *Lactococcus garvieae* are of particular interest due to their rapid acidification capacity, competitive fitness, and tolerance to environmental stress factors. According to recent studies, *Lactiplantibacillus plantarum* is considered one of the most effective bacterial inoculants for improving silage quality, particularly under low-temperature conditions [12,13,17]. In our study, the *Lactiplantibacillus plantarum* 29/9 strain demonstrated high adaptability to the ensiling of hard-stemmed raw materials (wheatgrass) under dry conditions, providing a stable pH reduction even at the highest NaCl concentration and antagonist activity, which is consistent with literature data. *Pediococcus* species such as *P. pentosaceus* and *P. acidilactici* are also often used in combination with other LAB for ensiling [34].

16S rRNA sequencing revealed the presence of *Lactococcus garvieae*, *Enterococcus faecalis*, and *Enterococcus faecium* among the active strains. The inclusion of representatives of the *Enterococcus* and *Lactococcus* genera (strains 28/8, 8/4, 5/3) in the formulation of developed bioproducts requires strict confirmation of their biotechnological safety. According to current criteria of the European Food Safety Authority (EFSA), natural strains of *E. faecium* and *E. faecalis* isolated from natural habitats and ensiled mass differ fundamentally from hospital isolates in the absence of pathogenicity determinants and clinical antibiotic resistance. During the ensiling process, they play a key ecological role as fermentation initiators, utilizing oxygen and lowering the pH at the initial stage. Enzymatic activity screening (Table 3) confirmed safety of the studied cultures of *E. faecalis* 28/8, *E. faecium* 8/4, and *L. garvieae* 5/3, as evidenced by the complete absence of ammonia production. This ensures that their metabolic activity is not associated with protein degradation to toxic biogenic amines (putrescine, cadaverine) or alkalization of the environment, and casein hydrolysis is limited, increasing the bioavailability of amino acids. Thus, the utilitarian potential of the strains outweighs the risks, and their safety is fully confirmed by the absence of undesirable volatile metabolites, making them valid components of starter cultures for harvesting wheatgrass. The presence of *Enterococcaceae* in silage and the use of inoculants from the genera *Enterococcus* and *Lactococcus* in silage have already been demonstrated previously [34,38,39,40]. In particular, the ability of *Enterococcus* sp. to produce lignocellulolytic enzymes was noted [34], which is also consistent with our studies—two of the three strains that actively used cellulose or hemicellulose were classified as belonging to the genus *Enterococcus*.

Only catalase-negative microbial isolates were selected for this study. Since *Staphylococcus* is typically catalase positive, its identification was unexpected in our study. However, there are published data on catalase-negative *Staphylococcus* species and strains [41,42,43]. However, the fact that isolate 5/4 belongs to this genus precludes its use as a starter microorganism, as it is not a LAB and may raise safety concerns.

The findings of the present study are consistent with current trends in silage biotechnology, which emphasize the use of indigenous LAB strains adapted to regional environmental conditions. Recent studies have demonstrated that the application of *Lactiplantibacillus plantarum*, *Pediococcus acidilactici*, and *Lactococcus garvieae* can improve silage fermentation characteristics, accelerate pH decline, enhance aerobic stability, and suppress undesirable microorganisms [34,44,45]. These findings support the potential use of the selected strains for the development of regionally adapted biological inoculants for difficult-to-ensile forage crops in northern Kazakhstan.

## 5. Conclusions

In this study, indigenous LAB associated with the epiphytic microbiota of *Agropyron* spp. and silage were isolated and comprehensively characterized. The selected strains exhibited desirable technological properties, including strong acidification capacity, antagonistic activity against undesirable microorganisms, and tolerance to osmotic stress. Molecular identification based on 16S rRNA gene sequencing revealed the predominance of *Lactococcus garvieae*, *Pediococcus acidilactici*, and *Lactiplantibacillus plantarum*, which are recognized for their biotechnological importance in lactic acid fermentation processes.

The use of regionally adapted strains may contribute to improved fermentation efficiency and nutrient preservation compared with conventional commercial inoculants. The findings demonstrate the high adaptive potential of indigenous LAB strains and allow us to select promising candidate strains for ensiling difficult-to-ensilage crops under the environmental conditions of northern Kazakhstan.

Future studies should focus on evaluating the performance of individual strains and strain consortia under laboratory-scale and industrial-scale ensiling conditions, as well as assessing their effects on silage quality, aerobic stability, and nutritional value.

## Figures and Tables

**Table 1:** Growth characteristics, pH, and titratable acidity of the selected LAB strains under different temperature conditions.

Strain Number	Source of Isolation	pH				°T			
		20 °С	26 °С	37 °С	45 °С	20 °С	26 °С	37 °С	45 °С
1/1	whole-plant sample	4.70 ± 0.01	4.70 ± 0.01	4.61 ± 0.01	4.71 ± 0.01	99.7 ± 1.5	99.7 ± 1.5	110.0 ± 1.7	100.3 ± 2.1
4/2	whole-plant sample	4.79 ± 0.02 ^a^	4.71 ± 0.02 ^ab^	4.49 ± 0.01 ^b^	–	95.3 ± 1.5 ^a^	100.3 ± 1.5 ^ab^	118.7 ± 1.5 ^b^	–
5/3	whole-plant sample	5.43 ± 0.02 ^a^	4.65 ± 0.01 ^ab^	3.99 ± 0.02 ^b^	4.48 ± 0.02 ^ab^	49.7 ± 1.5 ^a^	95.0 ± 1.0 ^ab^	119.3 ± 1.2 ^b^	120.3 ± 1.5 ^b^
8/4	whole-plant sample	4.61 ± 0.02 ^a^	4.49 ± 0.01 ^ab^	4.47 ± 0.02 ^b^	4.49 ± 0.01 ^ab^	110.7 ± 1.2	119.7 ± 1.5	120.0 ± 1.0	118.7 ± 1.5
12/5	silage	4.60 ± 0.02	4.62 ± 0.01	4.51 ± 0.02	4.61 ± 0.02	110.0 ± 1.0	110.0 ± 1.7	120.0 ± 1.0	110.3 ± 1.5
25/11	silage	4.51 ± 0.02 ^ab^	4.41 ± 0.02 ^ab^	4.22 ± 0.01 ^b^	4.61 ± 0.02 ^a^	119.7 ± 1.5 ^ab^	125.0 ± 1.0 ^ab^	130.3 ± 0.6 ^b^	100.0 ± 1.0 ^a^
26/6	silage	4.63 ± 0.02 ^a^	4.51 ± 0.02 ^ab^	4.39 ± 0.01 ^b^	4.60 ± 0.01 ^ab^	110.0 ± 1.7 ^a^	119.7 ± 2.1 ^ab^	125.0 ± 1.0 ^b^	109.7 ± 1.5 ^a^
27/7	silage	4.49 ± 0.02 ^ab^	4.38 ± 0.01 ^ab^	4.31 ± 0.02 ^b^	4.61 ± 0.01 ^a^	120.0 ± 2.0 ^ab^	125.0 ± 1.0 ^ab^	129.7 ± 2.1 ^b^	110.0 ± 1.0 ^a^
28/8	silage	4.54 ± 0.01 ^a^	4.41 ± 0.01 ^ab^	4.29 ± 0.02 ^b^	4.52 ± 0.01 ^ab^	119.3 ± 2.1 ^a^	125.0 ± 1.7 ^ab^	129.7 ± 1.5 ^b^	120.3 ± 1.5 ^a^
29/9	silage	4.61 ± 0.02 ^a^	4.60 ± 0.01 ^a^	4.43 ± 0.02 ^ab^	4.39 ± 0.02 ^b^	110.3 ± 1.5 ^a^	109.7 ± 1.2 ^a^	150.0 ± 2.0 ^b^	115.0 ± 1.0 ^ab^
30/10	silage	4.50 ± 0.02 ^ab^	4.49 ± 0.01 ^ab^	4.45 ± 0.01 ^b^	4.71 ± 0.02 ^a^	119.7 ± 1.5 ^ab^	120.3 ± 1.5 ^ab^	125.3 ± 1.5 ^b^	100.3 ± 2.3 ^a^
39/12	seed	4.51 ± 0.02 ^ab^	4.50 ± 0.01 ^ab^	4.31 ± 0.02 ^b^	4.84 ± 0.01 ^a^	120.3 ± 1.5 ^ab^	120.0 ± 1.0 ^ab^	130.0 ± 1.0 ^b^	95.3 ± 1.2 ^a^
41/13	spike	4.90 ± 0.01 ^ab^	4.80 ± 0.02 ^ab^	4.68 ± 0.01 ^b^	4.93 ± 0.01 ^a^	100.0 ± 1.0	99.7 ± 0.6	110.3 ± 1.5	101.3 ± 2.1
46/14	root	4.50 ± 0.02	4.50 ± 0.02	4.25 ± 0.02	–	120.0 ± 2.6	120.3 ± 1.5	139.7 ± 1.5	–
48/15	root	4.61 ± 0.02 ^a^	4.49 ± 0.01 ^ab^	4.32 ± 0.02 ^b^	–	110.0 ± 1.0 ^a^	120.3 ± 2.5 ^ab^	130.3 ± 1.2 ^b^	–

Note: Different superscript letters within the same row and parameter indicate statistically significant differences between cultivation conditions for the corresponding strain (Kruskal–Wallis test followed by Dunn’s post hoc test with Bonferroni correction, *p* < 0.05). The designation “ab” indicates an intermediate group that does not differ significantly from groups marked with the letters “a” and “b”. The symbol “–“ indicates the absence of strain growth under the corresponding cultivation conditions.

**Table 2:** Ability of the strains to ferment sugars and a polyhydric alcohol.

Strain No.	Glucose	рН	Xylose	рН	Fructose	рН	Maltose	рН	Lactose	рН	Raffinose	рН	Ribose	рН	Arabinose	рН	Rhamnose	рН	Mannose	рН	Cellulose	рН	Hemicellulose	рН	Starch	рН	Mannitol	рН
1/1	+++	4.11 ± 0.01	+++	4.12 ± 0.02	++	5.00 ± 0.02	+++	4.82 ± 0.02	+++	4.04 ± 0.01	+++	4.11 ± 0.02	+++	3.91 ± 0.01	+++	4.30 ± 0.02	+	6.20 ± 0.03	+++	4.52 ± 0.02	+	5.90 ± 0.03	+	6.19 ± 0.02	+++	4.20 ± 0.01	+++	4.05 ± 0.01
4/2	+++	4.13 ± 0.01	++	4.72 ± 0.03	++	4.72 ± 0.02	+	6.21 ± 0.03	+++	4.15 ± 0.02	+++	4.11 ± 0.01	+++	3.92 ± 0.01	++	4.90 ± 0.02	++	6.22 ± 0.03	+++	4.52 ± 0.02	+	6.00 ± 0.03	+	5.92 ± 0.02	+++	4.21 ± 0.01	+++	4.02 ± 0.01
5/3	+++	4.21 ± 0.02	+++	4.49 ± 0.01	+++	4.13 ± 0.02	+++	4.61 ± 0.02	++	5.02 ± 0.03	+	5.82 ± 0.03	+++	4.72 ± 0.02	+++	4.63 ± 0.02	+	6.05 ± 0.03	+++	4.00 ± 0.01	+	5.81 ± 0.02	+	5.89 ± 0.03	++	4.91 ± 0.02	+++	4.71 ± 0.02
8/4	+++	4.20 ± 0.01	+++	4.50 ± 0.02	+++	4.25 ± 0.01	+++	4.52 ± 0.02	+++	4.42 ± 0.02	+	5.88 ± 0.03	+++	4.63 ± 0.02	+++	4.33 ± 0.01	+	6.24 ± 0.03	+++	4.11 ± 0.01	+++	4.80 ± 0.02	+	5.89 ± 0.03	++	5.05 ± 0.02	+++	4.72 ± 0.02
12/5	+++	4.51 ± 0.02	+++	4.81 ± 0.03	+++	4.58 ± 0.02	+++	4.73 ± 0.02	+++	4.50 ± 0.02	+++	4.67 ± 0.02	+++	4.51 ± 0.01	+++	4.51 ± 0.01	++	5.82 ± 0.03	+++	4.45 ± 0.02	+	6.02 ± 0.03	+	6.10 ± 0.03	++	5.40 ± 0.02	+++	4.70 ± 0.02
25/11	+++	4.28 ± 0.02	+++	4.41 ± 0.02	+++	4.01 ± 0.01	++	5.57 ± 0.03	+++	4.61 ± 0.02	+	6.01 ± 0.03	+++	4.41 ± 0.02	+++	4.08 ± 0.01	++	5.88 ± 0.03	+++	4.05 ± 0.01	+	6.10 ± 0.03	+	6.05 ± 0.03	+	6.00 ± 0.03	+	6.02 ± 0.03
26/6	+++	4.39 ± 0.02	+++	4.44 ± 0.02	+++	4.34 ± 0.01	+++	4.00 ± 0.01	++	5.03 ± 0.03	+	6.00 ± 0.03	+++	4.40 ± 0.02	++	5.19 ± 0.03	+++	4.90 ± 0.02	+++	4.10 ± 0.01	++	5.21 ± 0.02	+	5.80 ± 0.03	+	6.03 ± 0.03	+++	4.71 ± 0.02
27/7	+++	4.30 ± 0.02	+++	4.33 ± 0.01	+++	4.42 ± 0.02	+++	4.33 ± 0.02	+	5.58 ± 0.03	+	5.82 ± 0.02	+++	4.27 ± 0.01	+++	4.40 ± 0.02	+	6.00 ± 0.03	+++	4.34 ± 0.02	+	6.01 ± 0.03	+	5.80 ± 0.02	+	6.12 ± 0.03	+++	4.72 ± 0.02
28/8	+++	4.40 ± 0.02	+++	4.43 ± 0.02	+++	4.11 ± 0.01	+++	4.41 ± 0.02	++	5.03 ± 0.03	+	5.90 ± 0.03	+++	4.48 ± 0.02	+++	4.39 ± 0.02	+	6.02 ± 0.03	+++	4.12 ± 0.01	+	6.15 ± 0.03	+	6.13 ± 0.03	+	6.00 ± 0.03	+++	4.72 ± 0.02
29/9	+++	4.32 ± 0.02	+++	4.41 ± 0.02	+++	4.11 ± 0.01	+++	4.10 ± 0.01	+++	4.32 ± 0.02	+++	4.10 ± 0.01	+++	4.55 ± 0.02	+++	4.22 ± 0.01	+	6.01 ± 0.03	+++	4.13 ± 0.01	+	6.14 ± 0.03	+++	4.62 ± 0.02	+	6.01 ± 0.03	+++	4.73 ± 0.02
30/10	+++	4.52 ± 0.02	+++	4.58 ± 0.02	+++	4.12 ± 0.01	+++	4.30 ± 0.02	++	5.41 ± 0.03	+++	5.82 ± 0.03	+++	4.52 ± 0.02	++	5.51 ± 0.03	+	6.00 ± 0.03	+++	4.09 ± 0.01	+	6.10 ± 0.03	+++	4.60 ± 0.02	+	6.05 ± 0.03	+++	4.70 ± 0.02
39/12	+++	4.48 ± 0.02	+++	4.37 ± 0.02	+++	4.64 ± 0.02	+++	4.64 ± 0.02	+	6.03 ± 0.03	+	6.03 ± 0.03	++	4.90 ± 0.02	++	5.65 ± 0.03	+	6.03 ± 0.03	+++	4.55 ± 0.02	−	−	−	−	−	−	−	−
41/13	+++	4.41 ± 0.02	+++	4.30 ± 0.01	+++	4.09 ± 0.01	+++	4.10 ± 0.01	+++	4.33 ± 0.02	+	5.70 ± 0.03	+++	4.60 ± 0.02	+++	4.22 ± 0.01	++	5.80 ± 0.03	+++	4.07 ± 0.01	+	6.10 ± 0.03	+	5.89 ± 0.03	−	5.89 ± 0.03	++	4.78 ± 0.02
46/14	+++	4.42 ± 0.02	+++	4.40 ± 0.02	+++	4.62 ± 0.02	+++	4.61 ± 0.02	+++	4.41 ± 0.02	+	5.91 ± 0.03	++	4.91 ± 0.02	++	5.20 ± 0.03	++	5.81 ± 0.03	++	4.81 ± 0.02	++	5.63 ± 0.03	++	4.87 ± 0.02	−	5.74 ± 0.03	+	6.12 ± 0.03
48/15	+++	4.34 ± 0.02	+++	4.41 ± 0.02	+++	4.41 ± 0.02	+++	4.44 ± 0.02	+++	4.51 ± 0.02	+++	4.62 ± 0.02	++	4.90 ± 0.02	++	5.20 ± 0.03	++	5.80 ± 0.03	+++	4.50 ± 0.02	++	5.62 ± 0.03	++	4.90 ± 0.02	−	5.72 ± 0.03	+	6.10 ± 0.03

Note: (−) no growth; (+) weak growth; (++) moderate growth; (+++) intensive growth. Values are presented as mean ± standard deviation (Mean ± SD) of three independent replicates.

**Table 3:** Proteolytic activity of the investigated strains.

Strains	Casein Proteolysis	Gelatin Proteolysis	Amylolytic Activity	Ammonia Production
1/1	−	−	−	−
4/2	−	−	−	−
5/3	+	−	−	−
8/4	+	−	−	−
12/5	++	−	−	−
25/11	++	−	−	−
26/6	+++	−	−	−
27/7	+++	−	−	−
28/8	+++	−	−	−
29/9	+	−	−	−
30/10	+++	−	−	−
39/12	++	−	−	−
41/13	+	−	−	−
46/14	+	−	−	−
48/15	++	−	−	−

Note: (−) no activity; (+) weak activity; (++) moderate activity; (+++) high activity.

**Table 4:** Salt tolerance characteristics of the selected strains at different NaCl concentrations.

Strain No.	log CFU/mL					pH					°T				
	NaCl Concentrations														
	2%	4%	6%	8%	10%	2%	4%	6%	8%	10%	2%	4%	6%	8%	10%
4/2	10.15 ± 0.16 ^a^	9.15 ± 0.16 ^ab^	9.00 ± 0.17 ^ab^	8.34 ± 0.17 ^ab^	7.04 ± 0.17 ^b^	5.51 ± 0.02 ^a^	5.60 ± 0.01 ^ab^	5.71 ± 0.03 ^ab^	5.83 ± 0.02 ^b^	5.83 ± 0.02 ^b^	55.3 ± 1.5 ^a^	50.3 ± 1.5 ^ab^	45.3 ± 1.5 ^ab^	40.3 ± 1.7 ^b^	40.3 ± 1.7 ^b^
5/3	11.36 ± 0.20 ^a^	10.00 ± 0.18 ^ab^	9.41 ± 0.18 ^ab^	5.30 ± 0.18 ^b^	–	5.40 ± 0.01 ^a^	5.51 ± 0.01 ^ab^	5.70 ± 0.02 ^ab^	5.93 ± 0.02 ^b^	–	60.3 ± 1.5 ^a^	55.0 ± 1.0 ^ab^	45.0 ± 2.0 ^ab^	35.3 ± 1.5 ^b^	–
8/4	10.20 ± 0.17 ^a^	10.08 ± 0.17 ^ab^	8.48 ± 0.17 ^ab^	6.75 ± 0.17 ^ab^	5.32 ± 0.17 ^b^	5.48 ± 0.02 ^a^	5.49 ± 0.01 ^a^	5.81 ± 0.01 ^ab^	5.72 ± 0.01 ^ab^	5.93 ± 0.02 ^b^	54.7 ± 1.5 ^a^	55.0 ± 2.0 ^a^	40.3 ± 1.7 ^ab^	45.3 ± 1.5 ^ab^	35.3 ± 1.5 ^b^
12/5	8.18 ± 0.15 ^a^	8.00 ± 0.15 ^ab^	7.65 ± 0.17 ^ab^	6.32 ± 0.16 ^b^	–	5.81 ± 0.01	5.81 ± 0.01	5.82 ± 0.02	5.74 ± 0.01	–	40.0 ± 1.0	40.0 ± 1.0	40.0 ± 1.0	45.3 ± 2.5	–
26/6	9.46 ± 0.17 ^a^	9.30 ± 0.18 ^ab^	8.30 ± 0.17 ^ab^	8.28 ± 0.18 ^ab^	5.04 ± 0.16 ^b^	5.59 ± 0.02 ^a^	5.70 ± 0.02 ^ab^	5.80 ± 0.02 ^ab^	5.82 ± 0.01 ^ab^	6.02 ± 0.03 ^b^	50.3 ± 1.5 ^a^	45.3 ± 1.5 ^ab^	39.7 ± 1.5 ^ab^	40.0 ± 1.0 ^ab^	30.3 ± 1.5 ^b^
27/7	8.49 ± 0.18 ^a^	7.30 ± 0.18 ^ab^	7.00 ± 0.17 ^ab^	7.00 ± 0.16 ^ab^	5.00 ± 0.18 ^b^	5.80 ± 0.03 ^ab^	5.80 ± 0.02 ^ab^	5.81 ± 0.03 ^ab^	5.71 ± 0.01 ^a^	5.89 ± 0.03 ^b^	40.0 ± 2.0 ^ab^	40.3 ± 1.7 ^ab^	40.3 ± 1.7 ^ab^	45.0 ± 1.0 ^a^	35.0 ± 1.0 ^b^
28/8	10.20 ± 0.14 ^a^	10.04 ± 0.16 ^ab^	9.32 ± 0.18 ^ab^	7.15 ± 0.16 ^ab^	5.46 ± 0.18 ^b^	5.52 ± 0.02 ^a^	5.52 ± 0.01 ^a^	5.69 ± 0.01 ^ab^	5.70 ± 0.01 ^ab^	5.78 ± 0.02 ^b^	55.7 ± 1.5 ^a^	55.3 ± 1.5 ^a^	45.0 ± 1.0 ^ab^	45.0 ± 2.0 ^ab^	39.7 ± 1.5 ^b^
29/9	10.67 ± 0.17 ^a^	10.58 ± 0.18 ^ab^	9.30 ± 0.17 ^ab^	8.11 ± 0.18 ^ab^	6.30 ± 0.18 ^b^	5.49 ± 0.02 ^a^	5.49 ± 0.01 ^a^	5.63 ± 0.01 ^ab^	5.83 ± 0.01 ^ab^	5.89 ± 0.01 ^b^	55.0 ± 1.0 ^a^	55.0 ± 1.2 ^a^	50.3 ± 1.5 ^ab^	40.3 ± 1.7 ^ab^	35.3 ± 1.5 ^b^
30/10	10.59 ± 0.18 ^a^	10.56 ± 0.17 ^ab^	9.26 ± 0.17 ^ab^	4.11 ± 0.17 ^b^	–	5.49 ± 0.01 ^a^	5.51 ± 0.01 ^ab^	5.63 ± 0.01 ^ab^	6.02 ± 0.02 ^b^	–	55.0 ± 2.0 ^a^	50.0 ± 1.0 ^ab^	50.0 ± 2.0 ^ab^	30.3 ± 1.5 ^b^	–
25/11	10.70 ± 0.17 ^a^	10.60 ± 0.19 ^ab^	8.00 ± 0.17 ^ab^	7.08 ± 0.18 ^ab^	5.30 ± 0.17 ^b^	5.49 ± 0.02 ^a^	5.59 ± 0.02 ^ab^	5.59 ± 0.02 ^ab^	5.73 ± 0.03 ^b^	5.83 ± 0.01 ^b^	55.3 ± 1.5 ^a^	50.0 ± 2.0 ^ab^	50.7 ± 2.1 ^ab^	45.3 ± 1.5 ^ab^	40.3 ± 1.7 ^b^
39/12	10.60 ± 0.17 ^ab^	10.62 ± 0.19 ^a^	9.18 ± 0.18 ^ab^	7.00 ± 0.15 ^ab^	5.00 ± 0.14 ^b^	5.50 ± 0.02 ^ab^	5.50 ± 0.01 ^a^	5.64 ± 0.02 ^ab^	5.74 ± 0.01 ^ab^	5.79 ± 0.02 ^b^	55.7 ± 1.5 ^ab^	55.0 ± 1.2 ^ab^	55.3 ± 1.5 ^ab^	45.7 ± 1.5 ^ab^	39.7 ± 2.1 ^b^
41/13	10.59 ± 0.14 ^ab^	10.62 ± 0.14 ^a^	9.00 ± 0.14 ^ab^	7.00 ± 0.17 ^ab^	5.08 ± 0.16 ^b^	5.51 ± 0.02 ^a^	5.50 ± 0.02 ^a^	5.62 ± 0.01 ^ab^	5.72 ± 0.01 ^ab^	5.82 ± 0.01 ^b^	55.0 ± 1.0 ^a^	55.0 ± 1.0 ^a^	50.0 ± 1.0 ^ab^	45.0 ± 1.0 ^ab^	40.0 ± 1.0 ^b^
46/14	10.23 ± 0.16 ^ab^	10.30 ± 0.18 ^a^	9.85 ± 0.18 ^ab^	7.78 ± 0.18 ^ab^	5.11 ± 0.17 ^b^	5.60 ± 0.01 ^ab^	5.59 ± 0.01 ^ab^	5.51 ± 0.02 ^a^	5.73 ± 0.02 ^ab^	5.84 ± 0.01 ^b^	50.0 ± 1.0 ^ab^	50.3 ± 1.7 ^ab^	54.7 ± 2.1 ^a^	44.7 ± 1.5 ^ab^	40.0 ± 2.0 ^b^
48/15	10.30 ± 0.17 ^a^	9.26 ± 0.16 ^ab^	7.15 ± 0.16 ^ab^	5.74 ± 0.16 ^ab^	5.18 ± 0.17 ^b^	5.59 ± 0.03 ^a^	5.71 ± 0.02 ^ab^	5.82 ± 0.01 ^ab^	5.92 ± 0.03 ^b^	5.91 ± 0.02 ^b^	50.0 ± 2.0 ^a^	45.0 ± 2.0 ^ab^	40.0 ± 1.0 ^ab^	35.0 ± 1.0 ^b^	34.7 ± 1.5 ^b^

Note. Different letters within a row indicate statistically significant differences among NaCl concentrations (*p* < 0.05), whereas identical letters indicate the absence of significant differences. The designation “ab” indicates an intermediate group that does not differ significantly from groups marked with the letters “a” and “b”. Differences among treatments were evaluated using the Kruskal–Wallis test followed by Dunn’s post hoc test with Bonferroni correction.

**Table 5:** Quantitative analysis of DNA samples performed using a NanoDrop 2000 spectrophotometer.

No.	Sample Name	DNA Concentration, ng/µL	260/280
1	5/3	315	1.8
2	5/4	328	1.92
3	25/11	141	1.97
4	29/9	231.4	1.84
5	30/10	150	2.0
6	46/14	150	2.0
7	8/4	180.4	1.99
8	28/8	156.9	1.97
9	48/15	184.9	1.88

**Table 6:** Identification results based on 16S rRNA gene nucleotide sequence analysis using BLAST+ v 2.17.0 (http://blast.ncbi.nlm.nih.gov/, accessed on 24 June 2026).

No.	Name	Sequence	Scientific Name	Per. Ident	Acc. Len	Accession
1	5/3	AAACGAACGCTAATACCGCATAACAATGAGAATCGCATGATTCTTATTTAAAAGAAGCAATTGCTTCACTACTTGATGATCCCGCGTTGTATTAGCTAGTTGGTAGTGTAAAGGACTACCAAGGCGATGATACATAGCCGACCTGAGAGGGTGATCGGCCACACTGGGACTGAGACACGGCCCAGACTCCTACGGGAGGCAGCAGTAGGGAATCTTCGGCAATGGGGGCAACCCTGACCGAGCAACGCCGCGTGAGTGAAGAAGGTTTTCGGATCGTAAAACTCTGTTGTTAGAGAAGAACGTTAAGTAGAGTGGAAAATTACTTAAGTGACGGTATCTAACCAGAAAGGGACGGCTAACTACGTGCCAGCAGCCGCGGTAATACGTAGGTCCCAAGCGTTGTCCGGATTTATTGGGCGTAAAGCGAGCGCAGGTGGTTTCTTAAGTCTGATGTAAAAGGCAGTGGCTCAACCATTGTGTGCATTGGAAACTGGGAGACTTGAGTGCAGGAGAGGAGAGTGGAATTCCATGTGTAGCGGTGAAATGCGTAGATATATGGAGGAACACCGGAGGCGAAAGCGGCTCTCTGGCCTGTAACTGACACTGAGGCTCGAAG	*Lactococcus garvieae*	100.00%	1502	PV682628.1
2	5/4	GCAGTCGAGCGAACAGAYGAGGAGCTTGCTCCTCTGACGTTAGCGGCGGRGGRAGAGTAACACGTGGATAACCTACCTATAAGACTGGGATAACTTCGGGAAACCGGAGCTAATACCGGATAATATATTGAACCGCATGGTTCAATAGTGAAAGACGGTTTTGCTGTCACTTATAGATGGATCCGCGCCGCATTAGCTAGTTGGTAAGGTAACGGCTTACCAAGGCAACGATGCGTAGCCGACCTGAGAGGGTGATCGGCCACACTGGAACTGAGACACGGTCCAGACTCCTACGGGAGGCAGCAGTAGGGAATCTTCCGCAATGGGCGAAAGCCTGACGGAGCAACGCCGCGTGAGTGATGAAGGTCTTCGGATCGTAAAACTCTGTTATTAGGGAAGAACAAATGTGTAAGTAACTATGCACGTCTTGACGGTACCTAATCAGAAAGCCACGGCTAACTACGTGCCAGCAGCCGCGGTAATACGTAGGTGGCAAGCGTTATCCGGAATTATTGGGCGTAAAGCGCGCGTAGGCGGTTTTTTAAGTCTGATGTGAAAGCCCACGGCTCAACCGTGGAGGGTCATTGGAAACTGGAAAACTTGAGTGCRGAAGAGGAAAGTGGAATTCCATGTGTAGCGGTGAAATGCGCAGAGATATGGAGGAACACCAGTGGCGAAGGCGACTTC	*Staphylococcus* sp.	99.42%	887	KX356359.1
			*Staphylococcus epidermidis*	99.27%	1212	MT559278.1
3	25/11	AACTTCCGTTAATTGATCAGGACGTGCTTGCACTGAATGAGATTTAAACACGAAGTGAGTGGCGGACGGGTGAGTAACACGTGGGTAACCTGCCCAGAAGCAGGGGATAACACCTGGAAACAGATGCTAATACCGTATAACAGAGAAAACCGCCTGGTTTTCTTTTAAAAGATGGCTCTGCTATCACTTCTGGATGGACCCGCGGCGCATTAGCTAGTTGGTGAGGTAACGGCTCACCAAGGCGATGATGCGTAGCCGACCTGAGAGGGTAATCGGCCACATTGGGACTGAGACACGGCCAGACTCCTACGGGAGGCAGCAGTAGGGAATCTTCCACAATGGACGCAAGTCTGATGGAGCAACGCCGCGTGAGTGAAGAAGGGTTTCGGCTCGTAAAGCTCTGTTGTTAAAGAAGAACGAATGGGTGAGAGTAACTGTTCACCCAGTGACGGTATTTAACCAGAAAGCCACGGCTAACTACTGTGCCAGCAGCCGCGGTAATACGTAGGTGGCAAGCGTTATCCGGATTTATTGGGCGTAAAGCGAGCGCAGGCGGTCTTTTAAGTCTAATGTGAAAGCCTTCGGCTCAACCGAAGAAGTGCATTGGAAACTGGGAGACTTGAGTGCAGAAGAGGACAGTGGAACTCCATGTGTAGCGGTGAAATGCGTAGA	*Pediococcus acidilactici*	99.41%	1337	KC200014.1
4	29/9	GCTCAGGACGAACGCTGGCGGCGTGCCTAATACATGCAAGTCGAACGAACTCTGGATTGATTGGTGCTTGCATCATGATTTACATTTGAGTGAGTGGCGAACTGGTGAGTAACACGTGGGAAACCTGCCCAGAAGCGGGGGATAACACCTGGAAACAGATGCTAATACCGCATAACAACTTGGACCGCATGGTCCGAGCTTGAAAGATGGCTTCGGCTATCACTTTTGGATGGTCCCGCGGCGTATTAGCTAGATGGTGGGGTAACGGCTCACCATGGCAATGATACGTAGCCGACCTGAGAGGGTAATCGGCCACATTGGGACTGAGACACGGCCCAAACTCCTACGGAAAGCAGCAGTAGGGAATCTTCCACAATGGACGAAAGTCTGATGGA GCAACGCCGCGTGAGTGAAGAAGGGTTTCGGCTCGTAAAACTCTGTTGTTAAAGAAGAACATATCTGAGAGTAACTGTTCAGGTATTTAACCAGAAAGCCACGGCTAACTACGTGCCAGCAGCCGCGGTAATACGTAGGTGGCAAGCGTTGTCCGGATTTATTGGGCGTAAAGCGAGCGCAAGCGGTTTTTTAAGTCTGATGTGAAAGCCTTCGGCTCAACCGAAGAAGTGCATCGGAAACTGGGAAACTTGAGTGCAGAAGAGGACAGTGGAACTCCATGTGTAGCGGTGAAATGCGTAGATATATGGAAGAACACCAGTGGCGAAGGCGGCTGTCTGGTCTGTAACTGACGCTGAGGCTCGAAAGTATGGGTAGCAAACAGGATTAGATA	*Lactiplantibacillus plantarum*	99.50%	3219240	CP071449.1
5	30/10	GTACAAGGCCCGGGAACGTATTCACCGCGGCGTGCTGATCCGCGATTACTAGCGATTCCGGCTTCATGCAGGCGAGTTGCAGCCTGCAATCCGAACTGAGAGAAGCTTTAAGAGATTTGCATGACCTCGCGGTCTAGCGACTCGTTGTACTTCCCATTGTAGCACGTGTGTAGCCCAGGTCATAAGGGGCATGATGATTTGACGTCATCCCCACCTTCCTCCGGTTTGTCACCGGCAGTCTCGCTAGAGTGCCCAACTAAATGATGGCAACTAACAATAAGGGTTGCGCTCGTTGCGGGACTTAACCCAACATCTCACGACACGAGCTGACGACAACCATGCACCACCTGTCACTTTGTCCCCGAAGGGAAAGCTCTATCTCTAGAGTGGTCAAAGGATGTCAAGACCTGGTAAGGTTCTTCGCGTTGCTTCGAATTAAACCACATGCTCCACCGCTTGTGCGGGCCCCCGTCAATTCCTTTGAGTTTCAACCTTGCGGTCGTACTCCCCAGGCGGAGTGCTTAATGCGTTTGCTGCAGCACTGAAGGGCGGAAACCCTCCAACACTTAGCACTCATCGTTTACGGCGTGGACTACCAGGGTATCTAATCCTGTTTGCTCACCACGCTTTCGAGCCTCAGCGTCAGTTACAGACCAGAGAGCCGCCTTCGCCACTGGTGTTCCTCCATATATCTACGCATTTCA	*Enterococcus faecalis*	99.86%	1470	MN255510.1
6	46/14	ATCACCGCTACACATGGAGTTCCACTGTCCTCTTCTGCACTCAAGTCTCCCAGTTTCCAATGCACTTCTTCGGTTGAGCCGAAGGCTTTCACATTAGACTTAAAAGACCGCCTGCGCTCGCTTTACGCCCAATAAATCCGGATAACGCTTGCCACCTACGTATTACCGCGGCTGCTGGCACGTAGTTAGCCGTGGCTTTCTGGTTAAATACCGTCACTGGGTGAACAGTTACTCTCACCCACAATGTTCTTCTTTAACAACAGAGCTTTACGAGCCGAAACCCTTCTTCACTCACGCGGCGTTGACTCCATCAGACTTGCGTCCATTGTGGAAGATTCCCTACTGCTGCCTACCGTTAGGAGTCTGGGCCGTGTCTCAGTCCCAATGTGGCCGATTACCCTCTCAGGTCGGCTACGCATCATCGCCTTGGTGAGCCGTTACCTCACCAACTAGCTAATGCGCCGCG	*Pediococcus* *acidilactici*	98.71%	626	MT598209.1
7	8/4	TCCTGGCTCAGGACGAACGCTGGCGGCGTGCCTAATACATGCAAGTCGAACGCTTCTTTTTCCACCGGAGCTTGCTCCACCGGAAAAAGAGGAGTGGCGAACGGGTGAGTAACACGTGGGTAACCTGCCCATCAGAAGGGGATAACACTTGGAAACAGGTGCTAATACCGTATAACAATCAAAACCGCATGGTTTTGATTTGAAAGGCGCTTTCGGGTGTCGCTGATGGATGGACCCGCGGTGCATTAGCTAGTTGGTGAGGTAACGGCTCACCAAGGCCACGATGCATAGCCGACCTGAGAGGGTGATCGGCCACATTGGGACTGAGACACGGCCCAAACTCCTACGGGAGGCAGCAGTAGGGAATCTTCGGCAATGGACGAAAGTCTGACCGAGCAACGCCGCGTGAGTGAAGAAGGTTTTCGGATCGTAAAACTCTGTTGTTAGAGAAGAACAAGGATGAGAGTAACTGTTCATCCCTTGACGGTATCTAACCAGAAAGCCACGGCTAACTACGTGCCAGCAGCCGCGGTAATACGTAGGTGGCAAGCGTTGTCCGGATTTATTGGGCGTAAAGCGAGCGCAGGCGGTTTCTTAAGTCTGATGTGAAAGCCCCCGGCTCAACCGGGGAGGGTCATTGGAAACTGGGAGACTTGAGTGCAGAAGAGGAGAGTGGAATTCCATGTGTAGCGGTGAAATGCGTAGATATATGGAGGAACACCAGTGGCGAAGGCGGCTCTCTGGTCTGTAACTGACGCTGAGGCTCGAAAGCGTGGGGAGCAAACAGGATTAGATACCCTGGTAGTCCACGCCGTAAACGATGAGTGCTAAGTGTTGGAGGGTTTCCGCCCTTCAGTGCTGCAGCTAACGCA	*Enterococcus faecium*	100.00%	2737559	CP131006.1
8	28/8	TCCTCCCGAGTGCTTGCACTCAATTGGAAAGAGGAGAGGCGGACGGGTGAGTAACACGTGGGTAACCTACCCATCAGAGGGGGATAACACTTGAAAACAGGTGCTAATACCGCATAACAGTTTATGCCGCATGGCATAAGAGTGAAAGGCGCTTTCGGGTGTCGCTGATGGATGGACCCGCGGTGCATTAGCTAGTTGGTGAGGTAACGGCTCACCAAGGCCACGATGCATAGCCGACCTGAGAGGGTGATCGGCCACACTGGGACTGAGACACGGCCCAGACTCCTACGGGAGGCAGCAGTAGGGAATCTTCGGCAATGGACGAAAGACTGACCGAGCAACGCCGCGTGAGTGAAGAAGGTTTTCGGATCGTAAAACTCTGTTGTTAGAGAAGAACAAGGACGTTAGTAACTGAACGTCCCCTGACGGTATCTAACCAGAAAGCCACGGCTAACTACGTGCCAGCAGCCGCGGTAATACGTAGGTGGCAAGCGTTGTCCGGATTTATTGGGCGTAAAGCGAGCGCAGGCGGTTTCTTAAGTCTGATGTGAAAGCCCCCGGCTCAACCGGGGAGGGTCATTGGAAACTGGGAGACTTGAGTGCAGAAGAGGAGAGTGGAATTCCATGTGTAGCGGTGAAATGCGTAGATATATGGAGGAACACCAGTGGCGAAGGCGGCTCTCTGGTCTGTAACTGACGCTGAGGCTCGAAAGCGTGGGGAGCAAACAGGATTAGATACCCTGGTAGTCCACGCCGTAAACTATGAGTGCTAAGTGTTGGAGGGTTTCCGCCCTTCAGTGCTGCAGCAA	*Enterococcus faecalis*	99.63%	837	KF250764.1
9	48/15	CCCAGAAGCAGGGGATAACACCTGGAAACAGATGCTAATACCGTATAACAGAGAAAACCGCCTGGTTTTCTTTTAAAAGATGGCTCTGCTATCACTTCTGGATGGACCCGCGGCGCATTAGCTAGTTGGTGAGGTAACGGCTCACCAAGGCGATGATGCGTAGCCGACCTGAGAGGGTAATCGGCCACATTGGGACTGAGACACGGCCCAGACTCCTACGGGAGGCAGCAGTAGGGAATCTTCCACAATGGACGCAAGTCTGATGGAGCAACGCCGCGTGAGTGAAGAAGGGTTTCGGCTCGTAAAGCGCGGTTGTTAAAGAAGAACGTGGGTGAGAGTAACTGTTCACCCAGTGACGGTATTTAACCAGAAAGCCACGGCTAACTACGTGCCAGCAGCCGCGGTAATACGTAGGTGGCAAGCGTTATCCGGATTTATTGGGCGTAAAGCGAGCGCAGGCGGTCTTTTAAGTCTAATGTGAAAGCCTTCGGCTCAACCGAAGAAGTGCATTGGAAACTGGGAGACTTGAGTGCAGAAGAGGACAGTGGAACTCCAATGTAGCGGTGAAATGCGTAGATATATGGAAGAACACCAGTGGCGAAGGCGGCTGTCTGGTCTGTAACTGACGCTGAGGCTCGAAAGCATGGGTAGCGAACAGGATTAGATACCCTGGTAGTCCATGCCGTAAACGATGATTACTAAGTGTTGGAGGGTTTCCGCCCTT	*Pediococcus acidilactici*	99.45%	1218	OL988614.1

## Data Availability

Data are available from the corresponding author upon reasonable request.

## Abbreviations

The following abbreviations are used in this manuscript:

LAB	Lactic acid bacteria
rRNA	Ribosomal RNA
MRS	Man–Rogosa–Sharpe
PCR	Polymerase chain reaction
MPG	Meat peptone gelatin
MPB	Meat peptone broth
